# Test–retest and inter-rater reliability of the incremental shuttle walk test in healthy university students

**DOI:** 10.1007/s11845-026-04406-y

**Published:** 2026-04-18

**Authors:** Yasemin Acar, Gülsüm Tıkaç

**Affiliations:** Department of Physiotherapy and Rehabilitation, Faculty of Health Sciences, Siirt University, Kezer Yerleşkesi Veysel Karani Mah. Üniversite Cad. No:1, 56100 Merkez/Siirt, Türkiye

**Keywords:** Incremental shuttle walk test, Test–retest reliability, Inter-rater reliability, Functional exercise capacity, University students

## Abstract

**Background:**

Although the Incremental Shuttle Walk Test (ISWT) is commonly used in clinical and research settings, data on its reliability and measurement error indices remain insufficient.

**Aims:**

To investigate the test–retest and inter-rater reliability of the ISWT in healthy university students and to establish reference values for measurement error indices.

**Methods:**

Forty-nine participants were recruited, of whom 45 completed all testing procedures. Each participant performed four ISWTs—two administered by Rater 1 and two by Rater 2—across two sessions conducted 2–7 days apart. Test–retest and inter-rater reliability were assessed using intraclass correlation coefficients (ICC), standard error of measurement (SEM), minimal detectable change at the 95% confidence level (MDC_95_), and Bland–Altman analyses.

**Results:**

The mean ISWT distance was 638.44 ± 103.50 m and 646.67 ± 109.44 m for Rater 1 across sessions (p=0.255), and 621.11 ± 123.33 m and 654.00 ± 142.53 m for Rater 2 (*p* < 0.001). Test–retest reliability was good to excellent, with ICCs of 0.899 for Rater 1 and 0.873 for Rater 2. Corresponding SEM and MDC_95_ values were 33.83 m/93.71 m and 47.43 m/131.39 m, respectively. Inter-rater reliability was also good to excellent, with ICCs of 0.869 in Session 1 and 0.899 in Session 2. Bland–Altman analyses confirmed acceptable agreement.

**Conclusions:**

The ISWT demonstrated good to excellent test–retest and inter-rater reliability, providing novel reference values for measurement error indices. These findings support its use as a reliable tool for assessing functional exercise capacity in both clinical and research settings.

**Clinical trial registration:**

ClinicalTrials.gov (Identifier: NCT06638281).

## Introduction

Assessment of functional exercise capacity is critically important in both clinical practice and scientific research, as it enables evaluation of an individual’s physical performance, prediction of disease prognosis, and assessment of therapeutic effectiveness [1]. Cardiopulmonary exercise testing (CPET) is considered the gold standard for this purpose. Through the direct, non-invasive measurement of ventilation and expired gases, CPET provides accurate and reproducible estimates of cardiorespiratory fitness (CRF) and peak oxygen uptake (peak VO₂), which is widely recognized as the most reliable indicator of maximal aerobic capacity. However, CPET requires specialized equipment, trained personnel, and considerable time investment, limiting its accessibility in many clinical and field settings [1, 2]. Consequently, field tests such as the six-minute walk test (6MWT) and the incremental shuttle walk test (ISWT) are widely used due to their simplicity, low cost, and practicality.

The 6MWT allows individuals to walk at their own pace, reflecting a submaximal effort. In contrast, the ISWT is a standardized, externally paced test with progressive speed increments, requiring individuals to walk at increasing speeds in response to audio signals [3, 4]. During the test, participants walk back and forth along a 10-m course, continuing until maximal exertion or symptom limitation is reached. Previous studies have shown that the ISWT elicits higher heart rates and greater breathlessness than the 6MWT, reflecting a greater physiological load [4]. Moreover, ISWT performance correlates strongly with peak VO₂ measured during CPET (*r* = 0.88), supporting its validity as a marker of functional exercise capacity [5]. While both the ISWT and 6MWT provoke oxygen uptake responses comparable to CPET, the patterns differ: the ISWT mirrors the incremental increase in VO₂ observed during laboratory-based exercise testing, whereas the 6MWT demonstrates a steady-state VO₂ profile after approximately three minutes [3].

Originally developed to evaluate functional exercise capacity in patients with chronic airway obstruction [4], the ISWT has since been applied in diverse populations, including those with bronchiectasis, heart failure, interstitial lung disease, ankylosing spondylitis, stroke, and healthy individuals [2, 6–10]. The measurement properties of the ISWT—including validity, reliability, and responsiveness—have been investigated mainly in clinical groups, particularly those with chronic respiratory diseases. The ISWT has consistently demonstrated strong test–retest reliability, with intraclass correlation coefficients (ICCs) often exceeding 0.80 [3]. High reproducibility has also been reported in cardiac rehabilitation populations, with ICC values ranging from 0.80 to 0.98 [8, 11]. Furthermore, validity and reliability have been confirmed in non-cystic fibrosis bronchiectasis, peripheral arterial disease, lung cancer, interstitial lung disease, as well as healthy populations [6, 9, 10].

Reliability testing is fundamental to establishing the reproducibility of outcome measures. Test–retest reliability evaluates the stability of measurements over time when no real change is expected, while inter-rater reliability examines consistency across different assessors administering the same test [12]. High reliability ensures that observed changes reflect true differences rather than measurement error, thereby supporting accurate clinical decision-making and research validity. However, most existing studies have focused primarily on test–retest reliability, with limited attention given to inter-rater reliability, despite its importance when multiple assessors are involved. Moreover, the majority of evidence derives from clinical populations, with scarce data in healthy individuals. In particular, little is known about the reliability of the ISWT in healthy young adults, and to our knowledge, no previous study has simultaneously examined both test–retest and inter-rater reliability in this group. Additionally, agreement analyses such as Bland–Altman plots and indices of measurement error—including the standard error of measurement (SEM) and minimal detectable change (MDC)—have rarely been reported.

Accordingly, the aim of this study was to comprehensively evaluate the test–retest and inter-rater reliability of the ISWT in healthy university students. Furthermore, the study sought to examine agreement using Bland–Altman methods and to calculate measurement error indices (SEM and MDC), thereby establishing reference reliability values for this population.

## Methods

### Study design and participants

This cross-sectional reliability study was conducted at Siirt University, Türkiye, between November 2024 and February 2025. Ethical approval was obtained from the Siirt University Ethics Committee (Approval No.: 2024/05/01/06), and written informed consent was obtained from all participants. The study was registered at ClinicalTrials.gov (Identifier: NCT06638281).

Inclusion criteria were: (i) being an undergraduate student in the Faculty of Health Sciences, Department of Nursing at Siirt University; (ii) aged 18–26 years; (iii) ability to understand and communicate in Turkish; and (iv) voluntary participation. Exclusion criteria were: (i) having any chronic systemic, orthopedic, or neurological disease and (ii) unwillingness to continue participation.

### Sample size calculation

Sample size was determined based on the study by Walter et al. [13], considering a minimum acceptable ICC of 0.60, an expected ICC of 0.80, a 5% type I error, and 80% power. Accordingly, at least 39 participants were required. To account for possible dropouts, 49 participants were included in the present study.

### Procedures

All participants performed the Incremental Shuttle Walk Test (ISWT) according to the standardized protocol described by Singh et al. [4]. The test was conducted in a 10-m corridor, marked by two cones placed 0.5 m from each end. A shuttle was defined as one 10-m lap. Walking speed was set by an audio signal, starting at 0.5 m/s (level 1) and increasing by 0.17 m/s each minute up to a maximum of 2.37 m/s at level 12. Standardized instructions and verbal encouragement were provided throughout the test.

Physiological parameters (heart rate, blood pressure, oxygen saturation) and subjective symptoms (dyspnea, leg fatigue, and general fatigue, rated with the Borg Category-Ratio 10 [CR10] scale) were recorded at three time points: before the test, immediately after test completion, and following a 5-minute recovery period. On the CR10 scale, 0 represented “no symptom at all” and 10 represented “maximum intensity” [14].

The test was terminated if the participant was unable to maintain the required walking pace due to dyspnea or fatigue, or if the participant was more than 0.5 m from the cone at the time of the audio signal after being given one opportunity to catch up [4, 11]. The completed shuttles were recorded and the total distance was calculated in meters.

### Test–retest and inter-rater protocol

To assess test–retest and inter-rater reliability, two physiotherapists (Rater 1 [R1] and Rater 2 [R2]) administered the ISWT to each participant on two separate occasions. During the first session, R1 and R2 conducted the ISWT with the same participant on the same day, with at least 30 min between assessments to avoid fatigue. The second session was performed 2–7 days later under similar conditions, during which both raters again administered the test to the same participant. Thus, each participant completed a total of four ISWTs (two per rater across two sessions).

**Fig. 1:**
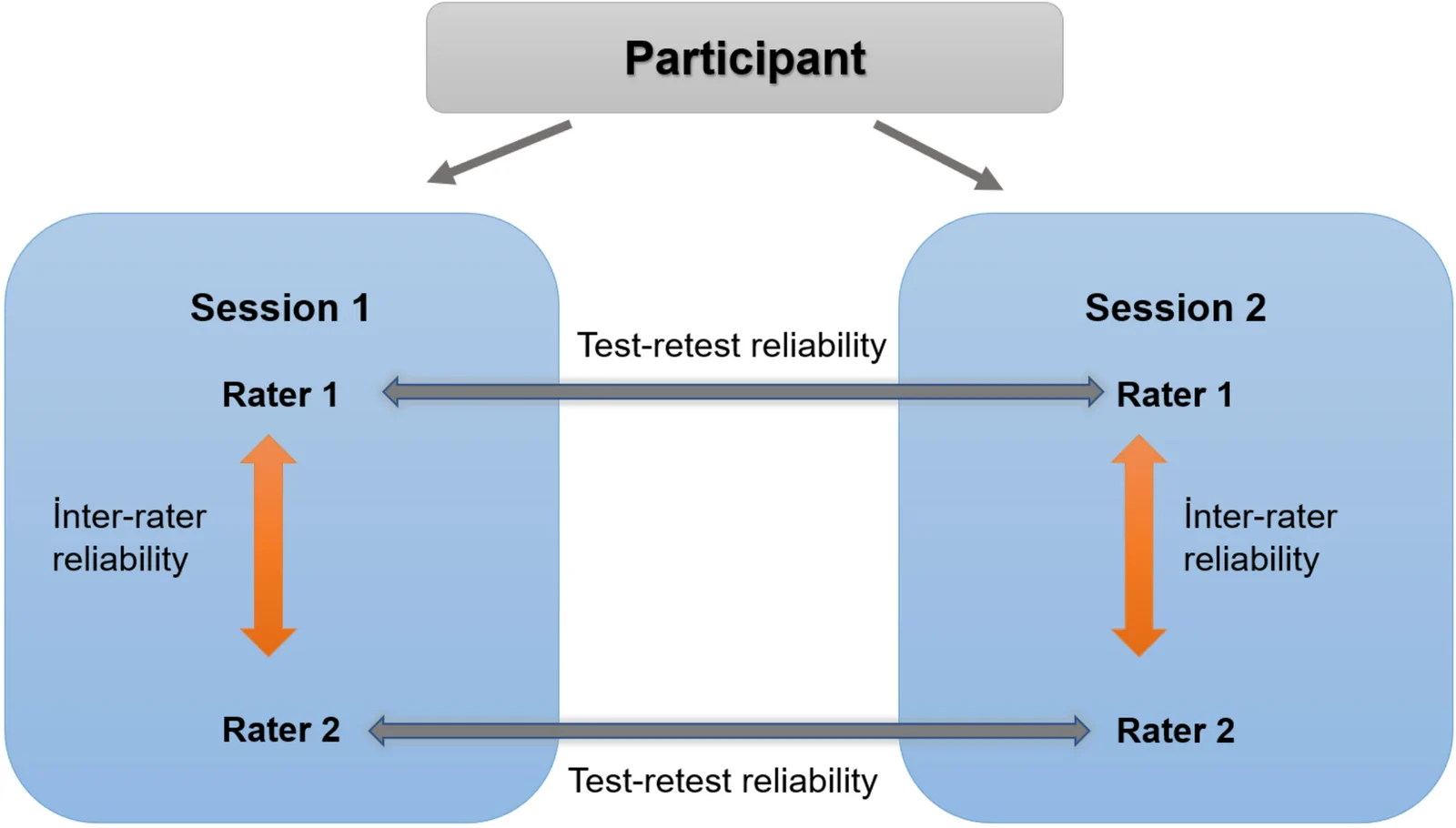
ISWT test–retest and inter-rater reliability protocol

Rater order was determined a priori using a computer-generated randomization list (Research Randomizer, www.randomizer.org*).* Accordingly, some participants were tested first by R1, others by R2; the same order was maintained in Session 2. Test–retest reliability was defined as repeated ISWTs by the same rater on different days, while inter-rater reliability referred to tests by different raters on the same day. The study protocol is illustrated in Fig. 1.

Prior to data collection, both raters reviewed the protocol to ensure consistency in test administration. R1 had prior experience with the ISWT, while R2 had not previously used it. Standardized instructions were provided to participants in all tests [3, 4]. Each rater recorded results independently, and data forms were stored separately to ensure blinding.

**Fig. 2:**
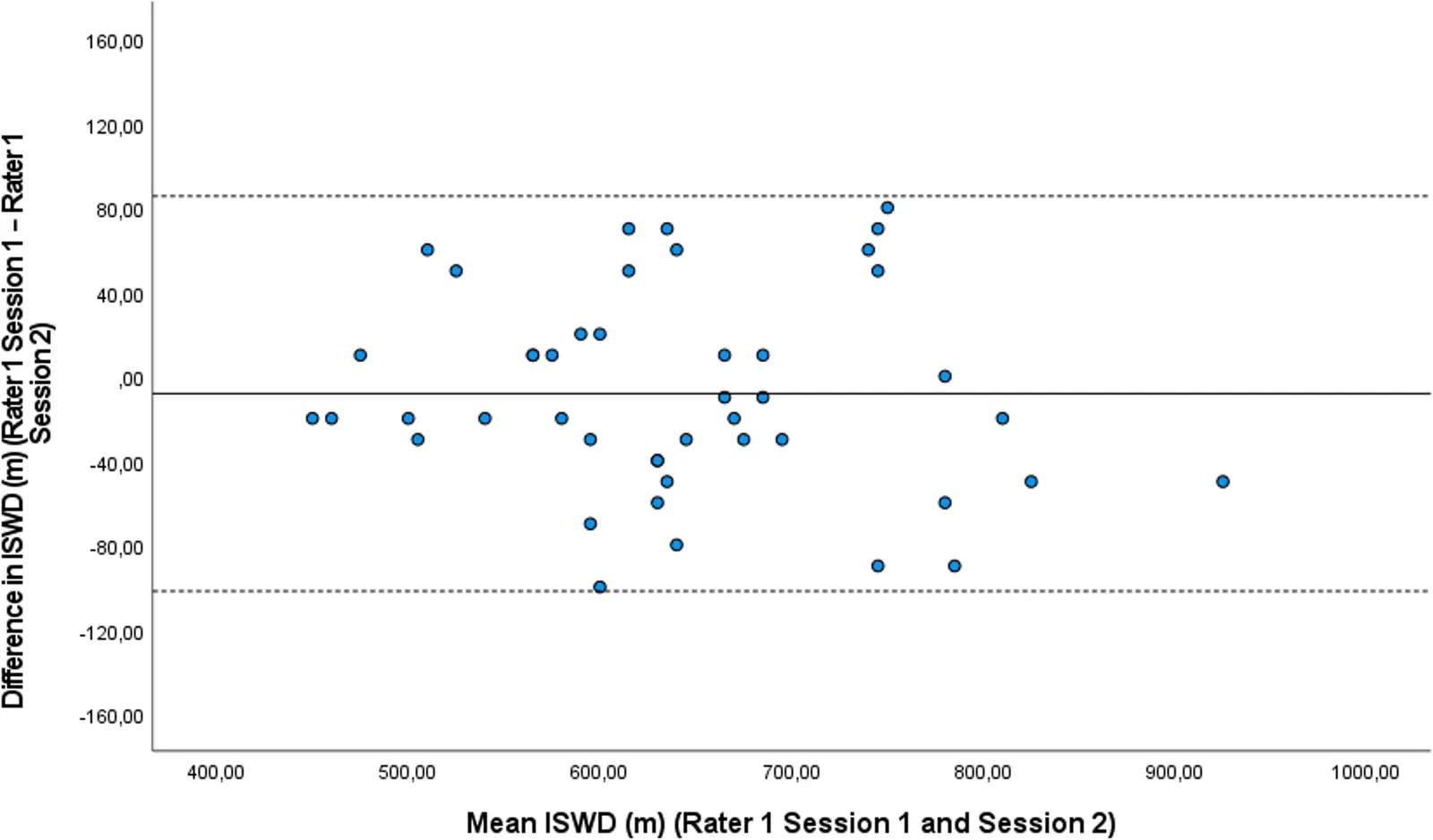
Bland–Altman plot for test–retest reliability (Rater 1). The plot shows the mean difference and 95% limits of agreement (LoA) between Session 1 and Session 2 measurements for Rater 1. The solid line represents the mean difference, and the dashed lines represent the upper and lower LoA

### Outcome measures

Baseline demographic and anthropometric characteristics (age, sex, height, weight, body mass index, and smoking status) were collected. The primary outcome was total distance walked during the ISWT. Secondary outcomes included heart rate, blood pressure, oxygen saturation, and CR10 scores for dyspnea, general fatigue, and leg fatigue.

**Fig. 3:**
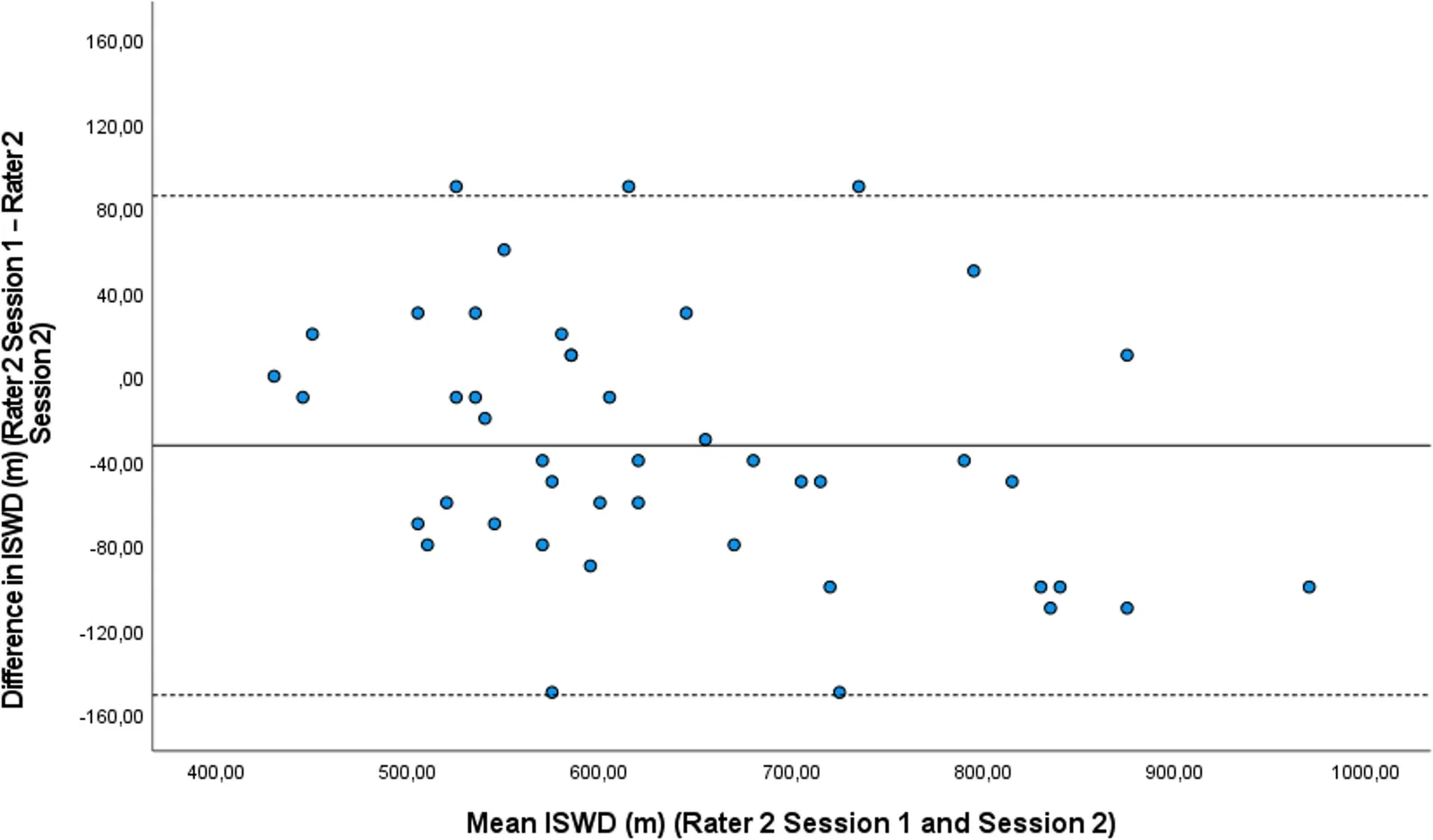
Bland–Altman plot for test–retest reliability (Rater 2). The plot shows the mean difference and 95% LoA between Session 1 and Session 2 measurements for Rater 2. The solid line indicates the mean difference, and the dashed lines indicate the LoA

### Statistical analysis

All statistical analyses were performed using IBM SPSS Statistics, version 22 (IBM Corp., Armonk, NY, USA). The normality of the data distribution was assessed using the Shapiro–Wilk test. Descriptive statistics were reported as mean ± standard deviation (SD) for continuous variables and as frequencies and percentages for categorical variables.

**Fig. 4:**
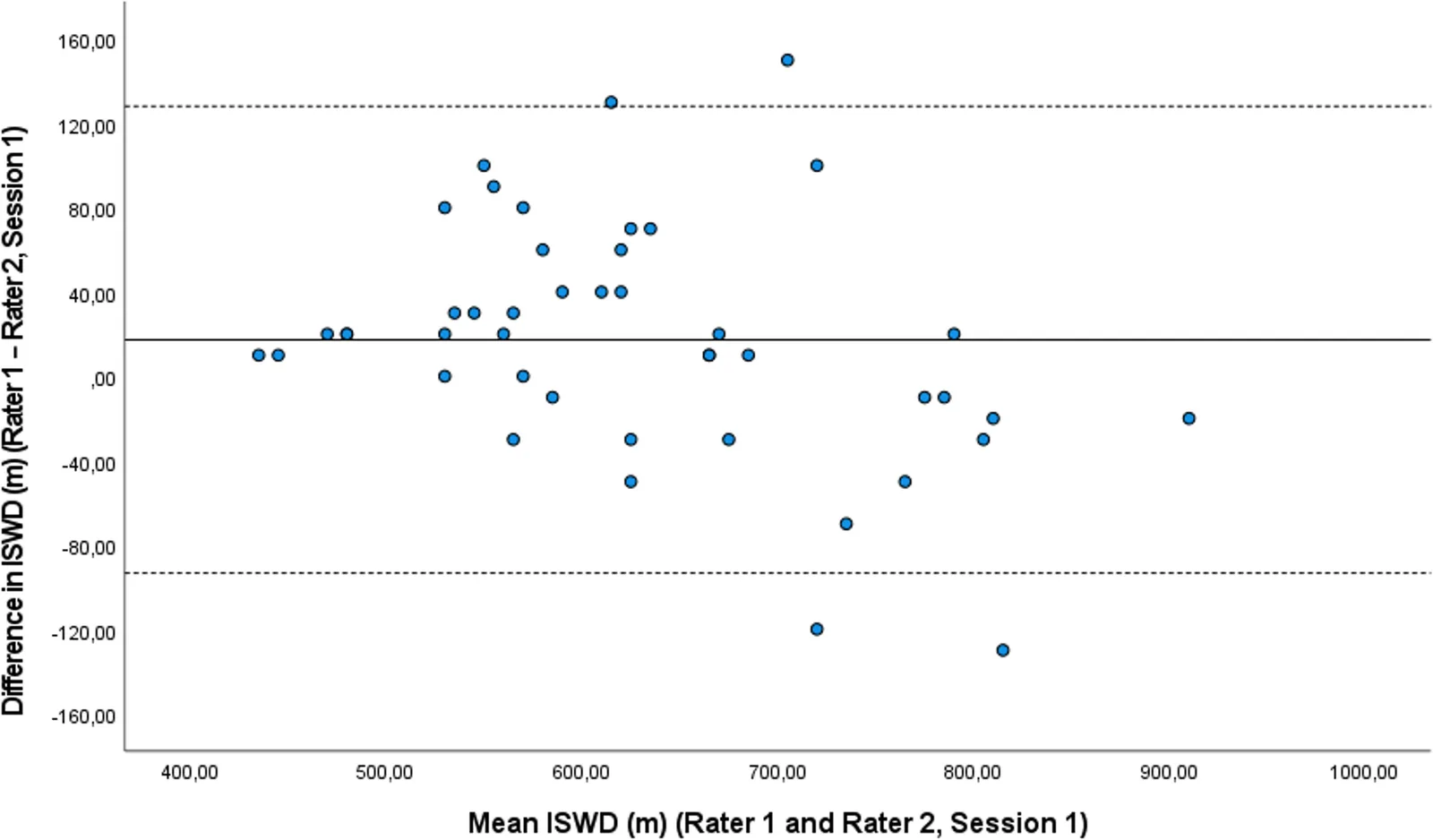
Bland–Altman plot for inter-rater reliability (Session 1: Rater 1 vs Rater 2). The plot illustrates the agreement between Rater 1 and Rater 2 in Session 1, with the mean difference (solid line) and 95% LoA (dashed lines)

Reliability was examined using intraclass correlation coefficients (ICCs) with 95% confidence intervals (CIs). A two-way mixed-effects model with absolute agreement [ICC(3,1)] was applied for both test–retest and inter-rater reliability. The ICC(3,1) model with absolute agreement was chosen because the same raters assessed all participants, making it appropriate for this design. Reliability strength was interpreted according to Koo and Li’s criteria [12], where ICC values < 0.50 indicate poor, 0.50–0.75 moderate, 0.75–0.90 good, and ≥ 0.90 excellent reliability.

Measurement error was quantified using the standard error of measurement (SEM), calculated as: SEM = SD_pooled_ × √(1 − ICC) where SD_pooled_​ represents the pooled standard deviation of the repeated measurements. The minimal detectable change (MDC) at the 95% confidence level was computed using the formula: MDC_95_ = SEM × 1.96 × √2 [15]. Agreement between measurements was further evaluated using Bland–Altman plots, in which the mean difference and 95% limits of agreement (mean difference ± 1.96 × SD of the differences) were calculated. The presence of systematic bias was assessed by testing whether the mean difference significantly differed from zero [16].

**Fig. 5:**
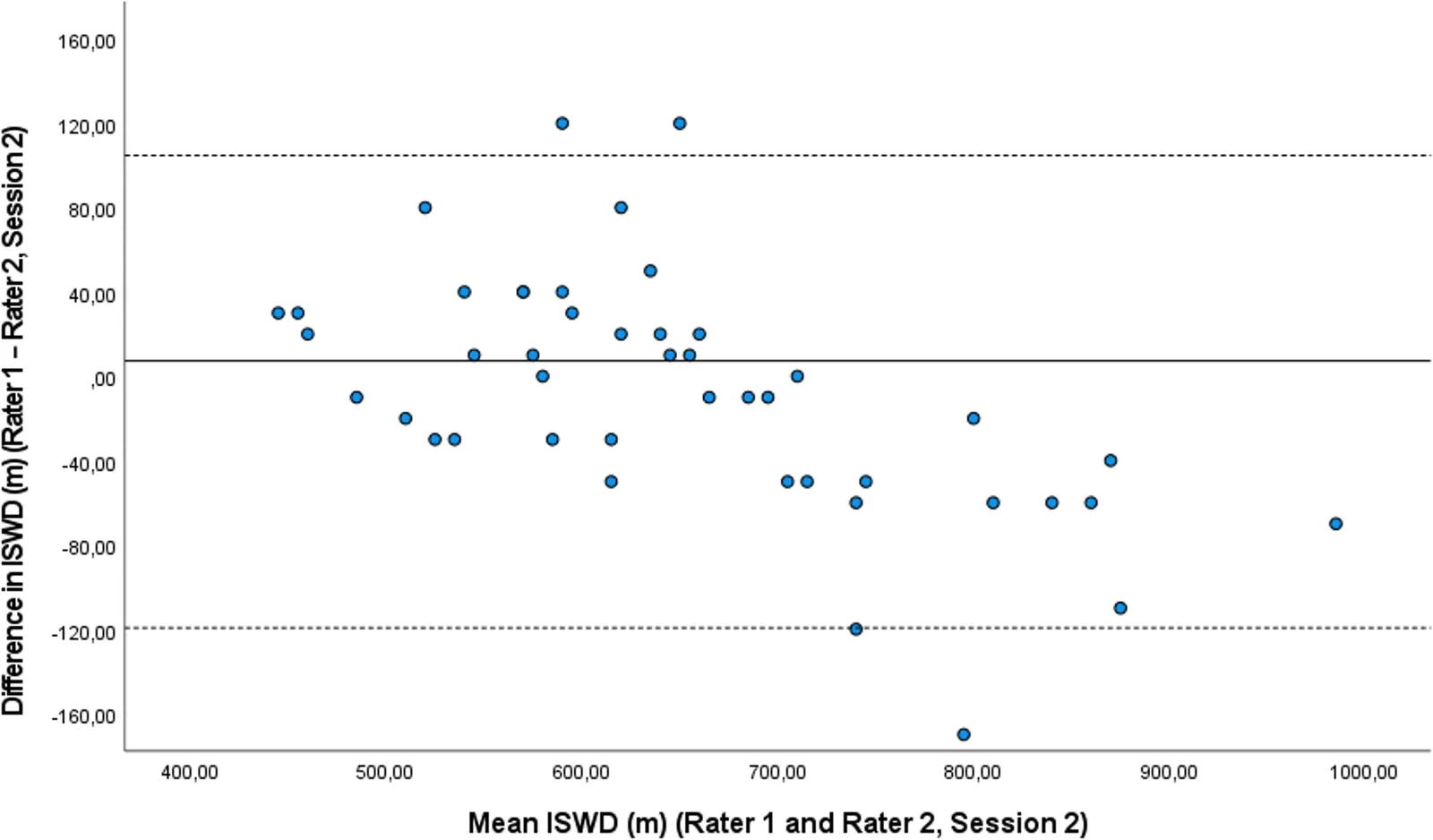
Bland–Altman plot for inter-rater reliability (Session 2: Rater 1 vs Rater 2). The plot illustrates the agreement between Rater 1 and Rater 2 in Session 2, with the mean difference (solid line) and 95% LoA (dashed lines)

A p-value < 0.05 was considered statistically significant.

## Results

A total of 49 participants were recruited, of whom 45 completed all testing procedures. The mean age was 21.33 ± 1.68 years; 29 (64.4%) were male and 16 (35.6%) were female. Baseline characteristics are presented in Table 1.

**Table 1:** Baseline characteristics of the participants (*n* = 45)

Variable	Mean ± SD	n (%)
Age, years	21.33 ± 1.68	
Sex		
Male		29 (64.4)
Female		16 (35.6)
Weight (kg)	65.80 ± 10.80	
Height (m)	1.71 ± 0.08	
BMI (kg/m²)	22.41 ± 3.05	
Smoking status		
Smokers		13 (28.9)
Non-smokers		32 (71.1)

*SD*, standard deviation; *kg,* kilogram; *m,* meter; *BMI,* body mass index

For Rater 1, the mean ISWT distance was 638.44 ± 103.50 m in the first session and 646.67 ± 109.44 m in the retest, with no significant difference between sessions (*p* = 0.255). For Rater 2, the mean ISWT distance increased from 621.11 ± 123.33 m to 654.00 ± 142.53 m (*p* < 0.001). Detailed ISWT results, including walking distance, physiological parameters, and symptom scores, are presented in Table 2.

**Table 2:** Test–retest results of the ISWT for Rater 1 and Rater 2 (*n* = 45)

	Rater 1 (Test / Retest)	p-value	Rater 2 (Test / Retest)	*p*-value
ISWT distance (m)	638.44 ± 103.50 / 646.67 ± 109.44	0.255	621.11 ± 123.33 /654.00 ± 142.53	<0.001
Heart rate (Pre-test)	88.91 ± 14.92 / 88.78 ± 14.20	0.947	92.87 ± 14.39 /89.62 ± 13.55	0.137
Heart rate (Post-test)	119.11 ± 19.35 / 120.18 ± 17.11	0.693	116.18 ± 19.76 /117.51 ± 21.28	0.637
SBP (Pre-test)	114.80 ± 10.14 / 115.87 ± 10.41	0.448	119.20 ± 10.04 /114.98 ± 11.95	0.005
SBP (Post-test)	136.91 ± 16.17 / 135.84 ± 16.45	0.645	136.96 ± 15.98 /137.51 ± 14.99	0.796
DBP (Pre-test)	68.91 ± 7.49 /68.51 ± 9.23	0.724	70.31 ± 9.28 /66.51 ± 9.77	0.018
DBP (Post-test)	76.31 ± 9.36 /75.64 ± 9.09	0.646	76.80 ± 12.18 /78.40 ± 13.14	0.535
SpO_2_ (Pre-test)	97.78 ± 1.24 /97.20 ± 1.78	0.054	97.60 ± 1.49 /97.60 ± 1.34	1.000
SpO_2_ (Post-test)	97.73 ± 1.37 /97.49 ± 1.59	0.306	97.16 ± 1.72 /97.49 ± 1.59	0.300
Dyspnea (Pre-test)	0.73 ± 0.89 /0.60 ± 0.96	0.244	0.67 ± 0.95 /0.49 ± 0.94	0.221
Dyspnea (Post-test)	4.09 ± 1.49 /3.56 ± 1.63	0.038	3.60 ± 2.24 /3.44 ± 2.00	0.564
General fatigue (Pre-test)	1.16 ± 1.09 /0.80 ± 0.91	0.044	1.22 ± 1.27 /0.91 ± 1.10	0.090
General fatigue (Post-test)	3.69 ± 1.59 /3.09 ± 1.56	0.009	3.69 ± 1.95 /3.07 ± 1.85	0.016
Leg fatigue (Pre-test)	0.80 ± 0.89 /0.87 ± 0.89	0.673	1.31 ± 1.41 /0.84 ± 0.97	0.014
Leg fatigue (Post-test)	4.62 ± 1.75 /4.24 ± 1.88	0.218	4.62 ± 2.16 /4.09 ± 1.73	0.037

*ISWT,* Incremental Shuttle Walk Test; *SBP,* Systolic Blood Pressure; *DBP,* Diastolic Blood Pressure; *SpO*_*2*_, Oxygen Saturation

Values are presented as mean ± SD

Test–retest comparisons were performed using the paired-samples t-test. *p* < 0.05 was considered statistically significant

### Test–retest reliability

The ISWT demonstrated good to excellent test–retest reliability. For Rater 1, the ICC was 0.899 (95% CI: 0.823–0.943), with an SEM of 33.83 m and an MDC_95_ of 93.71 m. For Rater 2, the ICC was 0.873 (95% CI: 0.716–0.937), with an SEM of 47.43 m and an MDC_95_ of 131.39 m. Bland–Altman analyses revealed mean differences of − 8.22 ± 47.82 m for Rater 1 and − 32.88 ± 60.43 m for Rater 2, with limits of agreement ranging from − 101.94 to 85.50 m and − 151.32 to 85.56 m, respectively **(**Table 3). The mean difference was not significant for Rater 1 (*p* = 0.255), whereas a systematic bias was observed for Rater 2 (*p* < 0.001). Bland–Altman plots for test–retest reliability are shown in Figs. 2 and 3.

**Table 3:** Test–retest and inter-rater reliability and agreement analyses of the ISWT

Comparison Type	Rater	Session	ISWT distance(m) ± SD	ICC (95% CI)	SEM (m)	MDC_95_ (m)	Mean Difference ± SD (m)	95% LoA (Lower, Upper)
Test–retest	Rater 1	Session 1	638.44 ± 103.50	0.899 (0.823–0.943)	33.83	93.71	-8.22 ± 47.82	-101.94, 85.50
		Session 2	646.67 ± 109.44					
Test–retest	Rater 2	Session 1	621.11 ± 123.33	0.873 (0.716–0.937)	47.43	131.39	-32.88 ± 60.43	-151.32, 85.56
		Session 2	654.00 ± 142.53					
Inter-rater	Rater 1	Session 1	638.44 ± 103.50	0.869 (0.769–0.927)	40.81	113.05	17.33 ± 56.50	-93.41, 128.07
	Rater 2	Session 1	621.11 ± 123.33					
Inter-rater	Rater 1	Session 2	646.67 ± 109.44	0.899 (0.825–0.943)	40.05	110.94	-7.33 ± 57.18	-119.40, 104.74
	Rater 2	Session 2	654.00 ± 142.53					

*ISWT,* Incremental Shuttle Walk Test; *ICC,* Intraclass Correlation Coefficient; *SEM,* Standard Error of Measurement; *MDC*_*95*_, Minimal Detectable Change at the 95% confidence level; *LoA*, Limits of Agreement

SEM was calculated as SD pooled × √(1 − ICC); MDC_95_ was calculated as SEM × 1.96 × √2. Mean difference and LoA values were derived from Bland–Altman analyses

### Inter-rater reliability

Inter-rater reliability was also good to excellent, with ICCs of 0.869 (95% CI: 0.769–0.927) in Session 1 and 0.899 (95% CI: 0.825–0.943) in Session 2. The SEM values were 40.81 m and 40.05 m, respectively. The MDC_95_ values were 113.05 m and 110.94 m, respectively. The Bland–Altman analyses indicated mean differences of 17.33 ± 56.50 m in Session 1 and − 7.33 ± 57.18 m in Session 2, with limits of agreement ranging from − 93.41 to 128.07 m, and − 119.40 to 104.74 m, respectively (Table 3). The Bland–Altman plots for inter-rater reliability are presented in Figs. 4 and 5.

## Discussion

This study is among the first to evaluate both test–retest and inter-rater reliability of the ISWT in healthy university students. Each participant completed four tests across two sessions administered by two different raters. Using ICCs, SEM, MDC_95_, and Bland–Altman analyses, our findings demonstrated good to excellent test–retest and inter-rater reliability of the ISWT in this population. These results extend the existing literature, which has predominantly focused on patient populations, by providing reference reliability data for healthy young adults.

When examining the walking distances, the mean ISWT distance of our participants (> 600 m) was consistent with normative values previously reported for healthy young adults. Agarwal et al. [17] reported average ISWT distances of 709.2 m in women and 807.9 m in men aged 17–40 years, while Azman et al. [10] found a median distance of approximately 780 m (interquartile range [IQR], 670–960 m) in healthy adults aged 21–39 years. The distances observed in our sample fall within these ranges, indicating that the functional capacity of our participants was comparable to that reported in healthy populations internationally.

### Test–retest reliability

The ISWT showed good to excellent test–retest reliability for both raters. ICC values were 0.899 (95% CI, 0.823–0.943) for Rater 1 and 0.873 (95% CI, 0.716–0.937) for Rater 2, confirming the stability of test results across repeated administrations over short intervals.

To date, only one study has specifically examined the test–retest reliability of the ISWT in a healthy population. Azman et al. [10] evaluated 199 healthy adults aged 21–80 years and reported an ICC of 0.91, indicating excellent repeatability between two trials conducted on the same day. The ICC value observed in that study aligns with our results, suggesting that the ISWT demonstrates stable performance not only in clinical but also in healthy populations.

The standard error of measurement (SEM) and the minimal detectable change (MDC) provide important information about the absolute reliability of the ISWT by quantifying measurement error and indicating the smallest amount of change that can be interpreted as real rather than random variation. The MDC, derived from the SEM, represents the minimum value that must be exceeded to be considered a true change beyond measurement error [18]. In the present study, Rater 1 demonstrated a SEM of 33.83 m with an MDC_95_ of 93.71 m, while Rater 2 demonstrated a SEM of 47.43 m with an MDC_95_ of 131.39 m. These values support the precision and stability of the ISWT when repeated in healthy young adults. Clinically, this means that an improvement greater than ~ 94 m (for Rater 1) or ~ 131 m (for Rater 2) can be interpreted as a real change in performance rather than measurement error.

Our findings are comparable to those reported in clinical populations. Quintino et al. [2] demonstrated good test–retest reliability of the ISWT in individuals with chronic stroke (ICC = 0.88; 95% CI, 0.78–0.93), with a SEM of 41.47 m and an MDC_95_ of 114.63 m. Singh et al. [9] further investigated the ISWT in patients with interstitial lung disease and reported excellent reliability (ICC = 0.91; 95% CI, 0.81–0.95), with a SEM of 19.5 m and an MDC_95_ of 53.9 m. The SEM and MDC values in ILD patients were lower than those in our study. This difference is likely due to the shorter walking distances in that population (~ 220 m on average) compared to the longer distances covered by our participants (> 600 m). Taken together, the SEM and MDC_95_ findings from our study reinforce the value of the ISWT as a sensitive tool for detecting meaningful changes in exercise capacity, while highlighting that absolute values may vary depending on population characteristics.

Bland–Altman analyses in our study showed that most participants fell within the calculated limits of agreement, indicating consistent performance across repeated ISWT administrations. However, the limits of agreement observed in our healthy participants were wider than that reported by Camargo et al. [6], who studied patients with non-cystic fibrosis bronchiectasis. This discrepancy is likely related to differences in study populations, testing intervals, and individual variations in motivation, suggesting that limits of agreement values can be influenced by both methodological and participant-related factors.

In the comparison between the two raters, Rater 1 demonstrated more favorable reliability results than Rater 2. ICC values were higher for Rater 1, which was supported by lower measurement error and smaller MDC_95_ values. In addition, Rater 1’s measurements showed narrower limits of agreement and no evidence of systematic bias. Although participants tended to walk longer distances in the second session for both raters, this systematic bias reached statistical significance only for Rater 2. These differences may be attributed to the fact that Rater 1 had prior experience administering the ISWT in both clinical and research contexts, whereas Rater 2 had not previously performed the test. These findings underscore the importance of adequate assessor training and standardized testing protocols to improve measurement accuracy and minimize variability in functional performance assessments such as the ISWT. They also highlight that even brief practical training or observation under an experienced assessor can enhance reliability, emphasizing the role of rater experience in both clinical practice and research settings.

Participants walked longer distances during the second session for both raters, suggesting the presence of a learning effect. This finding aligns with previous studies indicating that familiarization with the test procedure can lead to improved performance in subsequent trials. Hanson et al. [11] reported a significant improvement between the first and second ISWTs, attributing the increase in distance to test familiarization, although some studies in cardiac rehabilitation populations have found no significant change. Similarly, Azman et al. [10] observed greater distances in the second trial among healthy participants, further supporting the impact of learning. Singh et al. [4] also showed that patients tended to achieve greater distances in later attempts, and pooled data from Singh et al. [19] indicated a mean difference of around 20 m between the first two ISWTs. Taken together, these findings suggest that the improvement in ISWT distance observed in our study may be attributed to a learning effect. Although a familiarization trial was not conducted to avoid increasing the total number of tests per participant, the test procedure was explained in detail, and the order of raters for the first test was randomized. The absence of a familiarization trial may have slightly influenced the reliability indices, particularly for the novice rater. Future studies may consider including at least one familiarization trial to minimize learning effects and obtain more accurate reliability estimates. Therefore, when using the ISWT to monitor changes over time in clinical practice, at least two tests should be performed [3].

### Inter-rater reliability

The ISWT demonstrated good to excellent inter-rater reliability across both test sessions. In Session 1, the ICC was 0.869 (95% CI: 0.769–0.927), and in Session 2, the ICC increased to 0.899 (95% CI: 0.825–0.943), indicating strong agreement between raters. Absolute reliability indices further supported these findings. In Session 1, the SEM was 40.81 m with an MDC_95_ of 113.05 m. In Session 2, the SEM was slightly lower at 40.05 m, with a corresponding MDC_95_ of 110.94 m. These values suggest that an improvement greater than ~ 111–113 m can be interpreted as a real change beyond measurement error when the ISWT is administered by different raters. Bland–Altman analyses showed mean differences of 17.33 m in Session 1 and − 7.33 m in Session 2, with most of the observations falling within the calculated limits of agreement.

Only a limited number of studies have examined the inter-rater reliability of the ISWT, and all were conducted in clinical populations. Quintino et al. [2] reported excellent inter-rater reliability in individuals with chronic stroke (ICC = 0.93; SEM = 23.35 m; MDC = 64.53 m), while Wilkinson et al. [20] found similarly high reliability in patients with non-dialysis chronic kidney disease (ICC = 0.97; SEM = 7.1 m; MDC = 20 m). Both studies reported lower SEM and MDC values than our study, likely reflecting the shorter walking distances in their clinical samples (~ 400–560 m) compared with those in our healthy young participants (> 600 m). This highlights that absolute reliability indices such as SEM and MDC are influenced by population characteristics and performance levels. To our knowledge, no previous study has examined the inter-rater reliability of the ISWT in healthy individuals. Therefore, our study provides the first evidence demonstrating the reproducibility of the ISWT across different raters and offers novel reference values for healthy young adults.

### Clinical implications and future directions

These findings have important practical implications. The demonstrated reliability of the ISWT in healthy young adults supports its use as a simple, low-cost, and reproducible tool for assessing functional exercise capacity in non-clinical settings such as universities, sports science, and preventive health programs. Establishing reference values for measurement error indices further enhances the interpretability of ISWT results when monitoring changes over time. Future research should build on these results by including larger and more diverse samples and by examining potential subgroup differences according to sex, lifestyle factors, or physical activity levels.

### Limitations

This study has several limitations that should be considered when interpreting the results. First, the study sample was restricted to nursing students from a single university, which may limit the generalizability of the findings to the wider university student population. Being drawn from the same program and university, participants were relatively homogeneous not only in age and sex but also in education, health knowledge, motivation, and lifestyle factors. Therefore, caution is advised when applying these results to young adults from other universities, different fields of study, or to the general young adult population. Second, differences in rater experience may have contributed to the variability observed in inter-rater outcomes, underscoring the importance of adequate assessor training and protocol standardization to minimize measurement error.

## Conclusion

This study demonstrated that the Incremental Shuttle Walk Test (ISWT) exhibits good to excellent test–retest and inter-rater reliability in healthy university students. The intraclass correlation coefficients (ICCs), together with relatively low standard error of measurement (SEM) and minimal detectable change at the 95% confidence level (MDC_95_) values, confirm that the ISWT provides consistent and precise measurements when repeated over short intervals and when administered by different raters. Bland–Altman analyses further supported the agreement between assessments, with most observations falling within the calculated limits of agreement.

Importantly, these findings provide novel reference reliability values for healthy young adults, supporting the use of the ISWT as a reliable, practical, and reproducible tool for assessing functional exercise capacity in both research and clinical settings. To our knowledge, this is the first study to evaluate the inter-rater reliability of the ISWT in healthy young adults, thereby offering new evidence to guide future applications. Further research involving larger and more diverse student populations is warranted to confirm and extend these findings.

## Data Availability

Data are available from the corresponding author upon reasonable request.

## References

[1] Guazzi M, Adams V, Conraads V, Halle M, Mezzani A, Vanhees L, Arena R, Fletcher GF, Forman DE, Kitzman DW (2012) EACPR/AHA Joint Scientific Statement. Clinical recommendations for cardiopulmonary exercise testing data assessment in specific patient populations. Eur Heart J 33:2917–2927. 10.1093/eurheartj/ehs221 22952138

[2] Quintino LF, Aguiar LT, de Brito SAF, Pereira AS, Teixeira-Salmela LF, de Morais Faria CDC (2021) Reliability and validity of the incremental shuttle walking test in individuals after stroke. Top Stroke Rehabil 28:331–339. 10.1080/10749357.2020.1818481 32924882

[3] Holland AE, Spruit MA, Troosters T, Puhan MA, Pepin V, Saey D, McCormack MC, Carlin BW, Sciurba FC, Pitta F (2014) An official European Respiratory Society/American Thoracic Society technical standard: field walking tests in chronic respiratory disease. Eur Respir J 44:1428–1446. 10.1183/09031936.00150314 25359355

[4] Singh SJ, Morgan M, Scott S, Walters D, Hardman AE (1992) Development of a shuttle walking test of disability in patients with chronic airways obstruction. Thorax 47:1019–1024. 10.1136/thx.47.12.1019 1494764 PMC1021093

[5] Singh S, Morgan M, Hardman A, Rowe C, Bardsley P (1994) Comparison of oxygen uptake during a conventional treadmill test and the shuttle walking test in chronic airflow limitation. Eur Respir J 7:2016–2020. 10.1183/09031936.94.07112016 7875275

[6] De Camargo AA, Amaral TS, Rached SZ, Athanazio RA, Lanza FC, Sampaio LM, De Carvalho CR, Cukier A, Stelmach R, Dal Corso S (2014) Incremental shuttle walking test: a reproducible and valid test to evaluate exercise tolerance in adults with noncystic fibrosis bronchiectasis. Arch Phys Med Rehabil 95:892–899. 10.1016/j.apmr.2013.11.019 24361325

[7] Acar Y, İlçin N, Sarı İs, Önen F (2024) Functional exercise capacity in patients with ankylosing spondylitis. Physiother Theory Pract 40:2503–2509. 10.1080/09593985.2023.2263778 37776295

[8] Green D, Watts K, Rankin S, Wong P, O’Driscoll J (2001) A comparison of the shuttle and 6 minute walking tests with measured peak oxygen consumption in patients with heart failure. J Sci Med Sport 4:292–300. 10.1016/s1440-2440(01)80038-4 11702916

[9] Singh S, Moiz JA, Ali MS, Talwar D (2018) Reliability, validity, and responsiveness of the incremental shuttle walk test in patients with interstitial lung disease. J Cardiopulm Rehabil Prev 38:425–429. 10.1097/hcr.0000000000000327 29757823

[10] Azman MZB, Huang KS, Koh WJ, Leong SS, Ong B, Soon JL, Tan SW, Chan MY, Yang M, Yeung MT (2023) Normative reference values, determinants and regression equations for the incremental shuttle walk test (ISWT) in healthy Asian population aged 21 to 80 years. PLoS ONE 18:e0291132. 10.1371/journal.pone.0291132 37669286 PMC10479918

[11] Hanson LC, Taylor NF, McBurney H (2016) The 10 m incremental shuttle walk test is a highly reliable field exercise test for patients referred to cardiac rehabilitation: a retest reliability study. Physiotherapy 102:243–248. 10.1016/j.physio.2015.08.004 26538007

[12] Koo TK, Li MY (2016) A guideline of selecting and reporting intraclass correlation coefficients for reliability research. J Chiropr Med 15:155–163. 10.1016/j.jcm.2016.02.012 27330520 PMC4913118

[13] Walter S, Eliasziw M, Donner A (1998) Sample size and optimal designs for reliability studies. Stat Med 17(19980115):101–110. 10.1002/(sici)1097-0258. )17:1%3C101::aid-sim727%3E3.0.co;2-e 9463853

[14] Crisafulli E, Clini EM (2010) Measures of dyspnea in pulmonary rehabilitation. Multidiscip Respir Med 5:202. 10.1186/2049-6958-5-3-202 22958431 PMC3463047

[15] Weir JP (2005) Quantifying test-retest reliability using the intraclass correlation coefficient and the SEM. J Strength Cond Res 19:231–240. 10.1519/15184.1 15705040

[16] Bland JM, Altman DG (2010) Statistical methods for assessing agreement between two methods of clinical measurement. Int J Nurs Stud 47:931–936. 10.1016/j.ijnurstu.2009.10.001 2868172

[17] Agarwal B, Shah M, Andhare N, Mullerpatan R (2016) Incremental shuttle walk test: Reference values and predictive equation for healthy Indian adults. Lung India 33:36–41. 10.4103/0970-2113.173056 26933305 PMC4748663

[18] Haley SM, Fragala-Pinkham MA (2006) Interpreting change scores of tests and measures used in physical therapy. Phys Ther 86:735–743. 10.1093/ptj/86.5.735 16649896

[19] Singh SJ, Puhan MA, Andrianopoulos V, Hernandes NA, Mitchell KE, Hill CJ, Lee AL, Camillo CA, Troosters T, Spruit MA (2014) An official systematic review of the European Respiratory Society/American Thoracic Society: measurement properties of field walking tests in chronic respiratory disease. Eur Respir J 44:1447–1478. 10.1183/09031936.00150414 25359356

[20] Wilkinson TJ, Xenophontos S, Gould DW, Vogt BP, Viana JL, Smith AC, Watson EL (2019) Test–retest reliability, validation, and minimal detectable change scores for frequently reported tests of objective physical function in patients with non-dialysis chronic kidney disease. Physiother Theory Pract 35:565–576. 10.1080/09593985.2018.1455249 29601230

